# The role of anesthesiologists’ perceived self-efficacy in anesthesia-related adverse events

**DOI:** 10.1186/s12871-022-01732-3

**Published:** 2022-06-20

**Authors:** Feng Xu, Linlin Han, Shuai Zhao, Yafeng Wang, Qingtong Zhang, Erfeng Xiong, Shiqian Huang, Guixing Zhang, Hong He, Shiyu Deng, Yingjie Che, Yan Li, Liping Xie, Xiangdong Chen

**Affiliations:** 1 Department of Anesthesiology, Union Hospital, Tongji Medical College, Huazhong University of Science and Technology, Wuhan, 430022 Hubei China; 2 Department of Anesthesiology, The First Affiliated Hospital, School of Medicine, Shihezi University, Shihezi, 832002 China; 3 Department of Anesthesiology, The Third Affiliated Hospital, Sun Yat-Sen University, Guangzhou, 510630 China; 4 Department of Anesthesiology, Taihe Hospital, Hubei University of Medicine, Shiyan, 442000 China; 5 Department of Anesthesiology, Sichuan Academy of Medical Sciences & Sichuan Provincial People’s Hospital, Chengdu, 610072 China; 6 Department of Anesthesiology, The First Affiliated Hospital of Guangzhou Medical University, Guangzhou, 510120 China; 7 Department of Anesthesiology, The Eight Division Shihezi General Hospital, Shihezi, 832002 China

**Keywords:** Self-efficacy, Self-confidence, Anesthesia-related adverse events, Anesthesiologist, Anesthesia

## Abstract

**Background:**

Self-efficacy, as the vital determinant of behavior, influencing clinicians’ situation awareness, work performance, and medical decision-making, might affect the incidence of anesthesia-related adverse events (ARAEs). This study was employed to evaluate the association between perceived self-efficacy level and ARAEs.

**Methods:**

A cross-sectional study was performed in the form of an online self-completion questionnaire-based survey. Self-efficacy was evaluated via validated 4-point Likert scales. Internal reliability and validity of both scales were also estimated via Cronbach’s alpha and validity analysis. According to the total self-efficacy score, respondents were divided into two groups: normal level group and high level group. Propensity score matching and multivariable logistic regression were employed to identify the relationship between self-efficacy level and ARAEs.

**Results:**

The response rate of this study was 34%. Of the 1011 qualified respondents, 38% were women. The mean (SD) age was 35.30 (8.19) years. The Cronbach’s alpha of self-efficacy was 0.92. The KMO (KMO and Bartlett's test) value of the scale was 0.92. ARAEs occurred in 178 (33.0%) of normal level self-efficacy group and 118 (25.0%) of high level self-efficacy group. Before adjustment, high level self-efficacy was associated with a decreased incidence of ARAEs (RR [relative risk], 0.76; 95% CI [confidence interval], 0.62–0.92). After adjustment, high level self-efficacy was also associated with a decreased incidence of ARAEs (aRR [adjusted relative risk], 0.63, 95% CI, 0.51–0.77). In multivariable logistic regression, when other covariates including years of experience, drinking, and the hospital ranking were controlled, self-efficacy level (OR [odds ratio], 0.62; 95% CI, 0.46–0.82; *P* = 0.001) was significantly correlated with ARAEs.

**Conclusions:**

Our results found a clinically meaningful and statistically significant correlation between self-efficacy and ARAEs. These findings partly support medical educators and governors in enhancing self-efficacy construction in clinical practice and training.

**Supplementary Information:**

The online version contains supplementary material available at 10.1186/s12871-022-01732-3.

## Introduction

Self-efficacy theory is a key subset of Bandura’s social cognitive theory. According to this method, the vital determinants of behavior are self-efficacy and outcome expectancies [1, 2]. Self-efficacy is defined as the belief one holds in one’s ability to successfully execute a skill or behavior necessary for a desired outcome [3]. It is different from outcome expectancies, which refer to the perceived negative and positive consequences of performing a behavior. Self-efficacy is the central concept in numerous theories of behavior [4], and is able to highly predict a range of behaviors, such as work performance [5], academic performance [6], self-motivation [7], well-being [8], physical activity [9, 10], healthy eating [11], and health-related behaviors [12]. Accordingly, self-efficacy, as a self-perceived competence to succeed at a goal, is a robust predictor of outcomes.

Self-efficacy, is a crucial term to describe self-confidence and self-esteem, influencing clinicians’ performance and medical decisions for patients [13]. The majority of studies have been performed to measure and quantify self-efficacy in surgeons. Researchers have addressed a lack of self-efficacy of residents [13, 14], and have found that nearly 40% of residents reported low self-confidence in their skills after five years of training [15]. Obviously, lack of self-efficacy is considered a significant issue [16]. Thus, evaluating and examining the effect of self-efficacy on clinical practitioners is urgently needed.

Based on Bandura’s social cognitive theory and other studies [2, 17], high self-efficacy was proven to be related to more remarkable persistence, flexibility, and effort [18], and less self-distrust, self-doubt, and anxiety when confronting challenges [19]. In contrast, low self-efficacy may limit individuals' conviction and confidence to implement familiar techniques under stress. Within the anesthesia community, published studies have also reported that first-year clinical anesthesiology residents suffered from lower self-efficacy, and simulation boot camps increased self-efficacy and enriched self-confidence [19].

We thus deduced that self-efficacy differences in anesthesiologists may affect situational awareness and decision making, which can cause anesthesia-related adverse events (ARAEs). Accordingly, we assumed that self-efficacy grows with time and experience in clinical practice [20], and was associated with ARAEs to some extent. To quantify and measure self-efficacy, the general self-efficacy scale is internationally validated and has been utilized by numerous survey studies [21]. The purpose of this investigation was to examine the association between self-efficacy level and ARAEs. This work aimed to attract more attention to the effect of self-efficacy and promote a better understanding of its value in clinical practice.

## Methods

### Study design

An online self-completion questionnaire-based and anonymous survey was the basis of the study. The online survey did not include any personally identifiable information. The online survey study was also approved by the Chinese Association of Anesthesiologists (CAA).

**Fig. 1:**
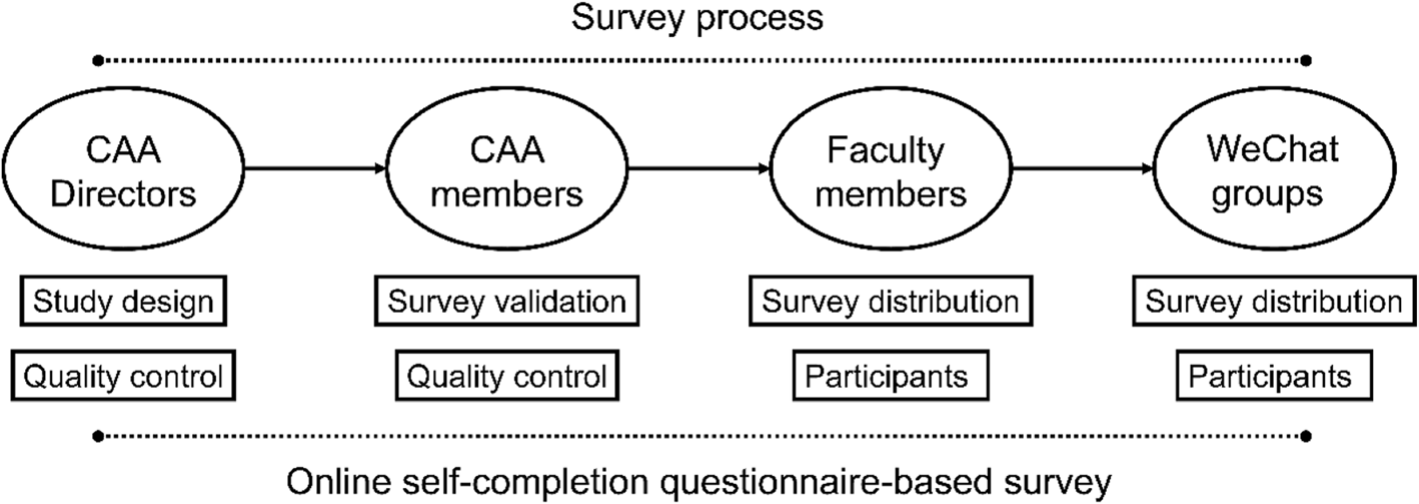
Online self-completion questionnaire-based survey. CAA, Chinese Association of Anesthesiologists

This survey was a cross-sectional study developed and distributed by directors of CAA. This manuscript adheres to the applicable STROBE guidelines. After validation by experts at CAA, emails were sent to CAA directors, requesting them to distribute the online survey link to faculty members and their particular WeChat groups (Fig. 1). Based on Bandura’s social cognitive theory and previous studies [21], we constructed a survey that included demographic characteristics, self-efficacy evaluation, educational background, academic performance, medical information, ARAE, etc. In general, after a brief assessment of the anesthesiologists’ self-efficacy, the survey concentrated on the relationship between self-efficacy and ARAEs. The online questionnaire was forwarded via the survey engine Wenjuanxing (https://www.wjx.cn/wjx/design/previewq.aspx?activity=115554789&s=1).

### Participants

This observational study was implemented in China from March 2021 to July 2021. Participants were all Chinese anesthesiologists and voluntarily completed the online survey. The answers were documented on the Wenjuanxing platform. The inclusion criteria were set as Chinese anesthesiologists, with the exception of interns and retirees. The online survey did not include any personally identifiable information. Hence, this study is exempt from ethical review. CAA has also granted a permission to this study.

### Survey selection

Using the same methods as those used in published studies [21], the general self-efficacy scale was used in this study. It is a validated and effective survey containing ten items, and each includes a 4-point Likert response option. Specifically, the general self-efficacy scale was as follows (Likert scale: 1 = not at all, 2 = hardly true, 3 = moderately true, 4 = exactly true): (1) I can always manage to solve difficult problems, if I try hard enough; (2) If someone opposes me, I can find the means and ways to get what I want; (3) It is easy for me to stick to my aims and accomplish my goals; (4) I am confident that I could deal efficiently with unexpected events; (5) Thanks to my resourcefulness, I know how to handle unforeseen situations; (6) I can solve most problems if I invest the necessary effort; (7) I can remain calm when facing difficulties because I can rely on my coping abilities; (8) When I am confronted with a problem, I can usually find several solutions; (9) If I am in trouble, I can usually think of a solution; (10) I can usually handle whatever comes my way.

### Self-efficacy level

The score for each item of the general self-efficacy scale was calculated for every participant, and the accumulation of their Likert scale responses was regarded as a final score. Higher scores indicated greater self-efficacy. This study aimed to examine the effect of perceived self-efficacy difference of anesthesiologists on influencing the incidence of ARAEs. According to the grade of the total self-efficacy score, we divided anesthesiologists into two groups: normal level (11–29) and high level (30–40) [21]. Whether anesthesiologists with high level of self-efficacy had lower ARARs incidence is a key question to be answered in this study.

### ARAEs

ARAE was defined as several adverse accidents during or after anesthesia. Main adverse events include [22, 23]: airway injury, nervous system injury, other injuries, airway management difficulties, intraoperative awareness, cardiac arrest, severe hypotension/hypertension, cardiocerebral events, severe hypoxia, life-threatening arrhythmia, severe allergy, blood transfusion complications, malignant hyperthermia, medication events, perioperative deaths, respiratory events, equipment issues, wrong sided procedures, severe complications caused anesthesia procedures (wrong medication), and other anesthesia-related accidents inducing damage to patients.

Anesthesiologists affirmed the occurrence of ARAEs based on the following: (1) recorded in the anesthesia note; (2) or in adverse events reporting system; or (3) recorded in case discussions. ARAEs occurred was recorded in detail through the self-reported online questionnaires, as shown in Table-S1.

### Outcome measurement

In our study, the primary outcome was a composite of ARAEs. The secondary outcome was the association between self-efficacy and ARAEs.

### Statistical analysis

Continuous variables of demographic data were recorded as the mean (standard deviation [SD]) or median (interquartile range [IQR]), such as age, body mass index (BMI), and working age. All other categorical variables were summarized with frequency and percentage, such as ARAE, gender, educational background, medical title, marital status, etc.

The internal consistency and reliability of the online survey were estimated using Cronbach’s alpha. Hotelling's T-squared test was used to determine the difference between items in scales. Validity analysis was implemented by KMO and Bartlett's test of sphericity. The value of the KMO measure of sampling adequacy (KMO value) was reported to describe the scale validity.

Without randomization in the survey study, anesthesiologist characteristics were imbalanced between the two groups. Therefore, we used a propensity score to account for possible confounding [24]. The propensity score was assessed via using multivariable logistic regression with self-efficacy level as the treatment variable. Each participant was weighted by the probability of that participant receiving the intervention that they did receive (the propensity score), which was analyzed by using the psmatch2 package in Stata. The nearest neighbor matching within caliper was used as the matching method, with the neighbor (1:1), replacement, and caliper (0.05). Ultimately, six samples in the normal level group did not support the matching and were removed. Then, 533 samples in each group were achieved after the nearest neighbor matching. In the weighted sample (the pseudo-sample) and unweighted sample, risk difference (RD) and relative risks(RR) were reported. The balance of covariates pre-and postweighting was evaluated by using standardized differences, and a standardized difference of ≥ 10% is a meaningful imbalance [25].

Multivariable logistic regression was employed to identify the potential risk factors for ARAEs. In binary logistic regression, Durbin-Watson test (DW) was performed to explore the independence among variables. VIF (variance inflation factor) was calculated to respond to the collinear statistics. Variables entry in model with *P* < 0.05 and variables removal in model with* P* > 0.05, were set in regression. The Hosmer and Lemeshow test was employed to evaluate the goodness of fit of the final binary logistic regression model. Odds ratios (ORs) and 95% confidence intervals (95% CIs) were reported to estimate the power of the association.

SPSS version 22.0 and Stata version 16.0 were used for all statistical analyses and graphing, and a *P*-value < 0.05 was regarded as statistically significant.

Concerning the sample size of this study, the sample size was calculated by the statistical formula of simple random sampling as follows:  n = Z^2^*P*(1-P)/e^2^. The confidence coefficient was set to 95%, with Z = 1.96. Due to no prior report of an accurate incidence of ARAE, *P* was set at a maximum of 0.5. The margin of error was set to 5%. Ultimately, approximately 385 respondents needed to be included in this study. Thus, 1011 respondents were sufficient, even concerning logistic regression.

## Results

### Participants’ characteristics

We have transferred this survey to 3016 anesthesiologists in 11 local anesthesiologists’ community WeChat groups. The response rate of this study was 34%. Ultimately, a total of 1011 anesthesiologists were enrolled in this study, including 627 men (62.02%) and 384 women (37.98%). The mean (SD) age of all respondents was 35.30 (8.19) years, ranging from 23 to 64 years. The baseline demographics were listed in Table 1. After weighting, participants’ characteristics were balanced in two groups, with a standardized difference of no more than 10%.

**Table 1:** Demographic characteristics of anesthesiologists with normal level or high level self-efficacy

	Unweighted (*N* = 1011)			Propensity score weighted^b^ (*N* = 1066)		
Characteristic	Normal level (*n* = 539)	High level (*n* = 472)	Std Diff (%)^c^	Normal level (*n* = 533)	High level (*n* = 533)	Std Diff (%)^c^
Age, y^a^			26.3			4.4
Mean (SD)	34.6 (7.7)	36.6 (7.8)		34.7 (7.8)	35.0 (7.7)	
Median (IQR)	33 (28, 39)	36 (30, 41)		33(28, 39)	33(28, 40)	
Years of experience, y			20.7			4.4
Mean (SD)	9.8 (8.6)	11.6 (8.5)		9.9 (8.6)	10.2 (8.5)	
Median (IQR)	7 (3, 15)	10 (5, 16)		7 (3, 15)	8 (3, 15)	
Gender, NO. (%)			-19.2			-2.3
Male	311 (57.7)	316 (67.0)		310 (58.2)	316 (59.3)	
Female	228 (42.3)	156 (33.0)		223 (41.8)	217 (40.7)	
Race, NO. (%)			-7.5			-3.9
Han	514 (95.4)	457 (96.8)		510 (95.7)	514 (96.4)	
Non-Han	25 (4.6)	15 (3.2)		23 (4.3)	19 (3.6)	
BMI, Kg/m^2^			16.4			5.4
Mean (SD)	22.7(2.9)	23.2 (3.0)		22.7 (2.9)	22.9 (3.2)	
Median (IQR)	22.6 (20.7, 24.5)	23 (21, 25)		22.7 (20.8, 24.7)	22.9 (21.0, 24.8)	
Medical title, NO. (%)			26.2			8.9
Resident anesthesiologist	259 (48.1)	157 (33.3)		254 (47.6)	227 (42.6)	
Attending anesthesiologist	180 (33.4)	204 (43.2)		179 (33.6)	197 (37.0)	
Deputy/chief anesthesiologist	100 (18.5)	111 (23.5)		100 (18.8)	109 (20.4)	
Hospital ranking where working, NO. (%)			17.2			1.4
III-A	362 (67.2)	281 (59.5)		357 (67.0)	356 (66.8)	
III-B	52 (9.6)	58 (12.3)		52 (9.8)	51 (9.5)	
II-A	92 (17.1)	86 (18.2)		92 (17.2)	89 (16.7)	
II-B	21 (3.9)	24 (5.1)		21 (3.9)	26 (4.9)	
I	12 (2.2)	23 (4.9)		11 (2.1)	11 (2.1)	
Smoking, NO. (%)			-8.7			-4.3
Yes	100 (18.6)	104 (22.0)		99 (18.6)	108 (20.3)	
No	439 (81.4)	368 (78.0)		434 (81.4)	425 (79.7)	
Drinking, NO. (%)			-8.1			7.3
Yes	226 (41.9)	217 (46.0)		224 (42.0)	205 (38.5)	
No	313 (58.1)	255 (54.0)		309 (58.0)	328 (61.5)	
Educational background, NO. (%)			-13.0			0.3
College degree	17 (3.1)	24 (5.1)		17 (3.2)	20 (3.8)	
Bachelor degree	304 (56.4)	286 (60.6)		302 (56.7)	302 (56.6)	
Master degree	197 (36.6)	144 (30.5)		196 (36.8)	186 (34.9)	
Doctor degree	21 (3.9)	18 (3.8)		18 (3.3)	25 (4.7)	
Overseas study experience, NO. (%)			-10.2			-0.9
Yes	22 (4.1)	30 (6.4)		22 (4.1)	23 (4.3)	
No	517 (95.9)	442 (93.6)		511 (95.9)	510 (95.7)	
Marriage, NO. (%)			-30.0			0.0
Yes	367 (68.1)	382 (80.9)		366 (68.7)	363 (68.1)	
No	66 (12.2)	40 (8.5)		66 (12.4)	72 (13.5)	
Lover	106 (19.7)	50 (10.6)		101 (18.9)	98 (18.4)	
Having children, NO. (%)			26.5			-0.4
Yes	316 (58.6)	336 (71.2)		315 (59.1)	314 (58.91)	
No	223 (41.4)	136 (28.8)		218 (40.9)	219 (41.1)	
Publishing scientific papers in English, NO. (%)			11.2			-5.7
Yes	74 (12.7)	84 (17.8)		72 (13.5)	62 (11.6)	
No	465 (86.3)	338 (82.2)		461 (86.5)	471 (88.4)	
Personal annual income/CNY, NO. (%)			21.5			6.0
< 10,000	71 (13.1)	38 (8.0)		69 (12.9)	60 (11.3)	
10,000–50,000	59 (11.0)	37 (7.8)		57 (10.7)	48 (9.0)	
50,000–100,000	184 (34.1)	154 (32.6)		182 (34.2)	213 (40.0)	
100,000–200,000	165 (30.6)	181 (38.4)		165 (31.0)	136 (25.5)	
200,000–300,000	45 (8.4)	48 (10.2)		45 (8.4)	52 (9.7)	
> 300,000	15 (2.8)	14 (3.0)		15 (2.8)	24 (4.5)	
Patients severity reported by participants (ASA grading)			-1.0			7.1
Participants reporting ARAEs	178	118		177	111	
Patients severity: ASA-I	12 (6.7)	10 (8.5)		12 (6.8)	13 (11.7)	
Patients severity: ASA-II	53 (29.8)	37 (31.3)		53 (30.0)	23 (20.7)	
Patients severity: ASA-III	113 (63.5)	71 (60.2)		112 (63.2)	75 (67.6)	

*Abbreviations*: *BMI* Body mass index, *SD* Standard deviation, *Std Diff* Standardized difference, *IQR* Interquartile range

^a^All variables are depicted as No. (%) except age, BMI and years of experience, which presented as mean (SD)/median (IQR)

^b^A pseudo-sample was obtained after weighting based on propensity score match

^c^Std Diff of less than 10% are considered to indicate good balance between groups

### Internal consistency reliability and validity

According to statistics, Cronbach’s alpha of 0.70 or over is considered to be acceptable, and a value of 0.90 or over is considered excellent [26]. Table 2 shows statistical indicators responding to internal consistency reliability and validity. In the general self-efficacy scale, Cronbach’s alpha was 0.92, and Cronbach's alpha based on standardized items was 0.92, which were all over 0.90 and demonstrated excellent internal consistency reliability. Hotelling's T-squared test found that the mean value of items in the scale was different, with *P* < 0.001. When referring to the validity of the scale, KMO and Bartlett's test of sphericity showed that the KMO value was 0.93 (*P* < 0.001), which indicated that validity was perfect (KMO value over 0.90).

**Table 2:** Assessment of internal consistency and validity of Likert scales

Statistics	General self-efficacy scale
Number of items	10
Hotelling's T-squared test, *P*-value	< 0.001
Cronbach's Alpha	0.92
Cronbach's Alpha based on standardized Items	0.92
KMO value	0.93
KMO and Bartlett's test of sphericity, *P*-value	< 0.001

The total validity of self-efficacy scale measured by KMO and Bartlett's test of sphericity; Cronbach's Alpha or KMO value of 0.7 (acceptable), 0.8 (good) and 0.9 (excellent)

### Main outcome

Among 1011 participants, 296 anesthesiologists experienced ARAEs during anesthesia, with an incidence of 29.28%. ARAEs occurred in 178 (33.0%) of normal level self-efficacy group and 118 (25.0%) of high level self-efficacy group, as shown in Table 3. Before adjustment, high level self-efficacy was associated with a decreased incidence of ARAEs (RR, 0.76; 95% CI, 0.62–0.92). After adjustment via propensity score match, high level self-efficacy was still associated with a decreased incidence of ARAEs (aRR, 0.63; 95% CI, 0.51–0.77).

**Table 3:** The incidence of ARAEs in anesthesiologists with normal level and high level self-efficacy

Characteristic	Unweighted (*N* = 1011)					Propensity score weighted^a^ (*N* = 1066)				
	Normal level (*n* = 539)	High level (*n* = 472)	RD	RR	*P*^*b*^	Normal level (*n* = 533)	High level (*n* = 533)	aRD	aRR	*P*^*b*^
Anesthesia-related adverse events, No. (%)			-0.08 (-0.14, -0.025)	0.76 (0.62, 0.92)	0.0052			-0.12 (-0.18, -0.071)	0.63 (0.51, 0.77)	< 0.001
Yes	178 (33.0)	118 (25.0)				177 (33.2)	111 (20.8)			
No	361(67.0)	354 (75.0)				356 (66.8)	422 (79.2)			

*Abbreviations*: *SD* Standard deviation, *IQR* Interquartile range, *CI* Confidence interval, *RR* Relative risk, *RD* Risk difference, *aRR* Adjusted relative risk, *aRD* Adjusted risk difference, *ARAEs* Anesthesia-related adverse events

^a^ A pseudo-sample was obtained after weighting based on propensity score match

^b^ Chi-square test used in exploring difference between two groups

### Multivariable logistic regression analysis of risk factors for ARAEs

**Fig. 2:**
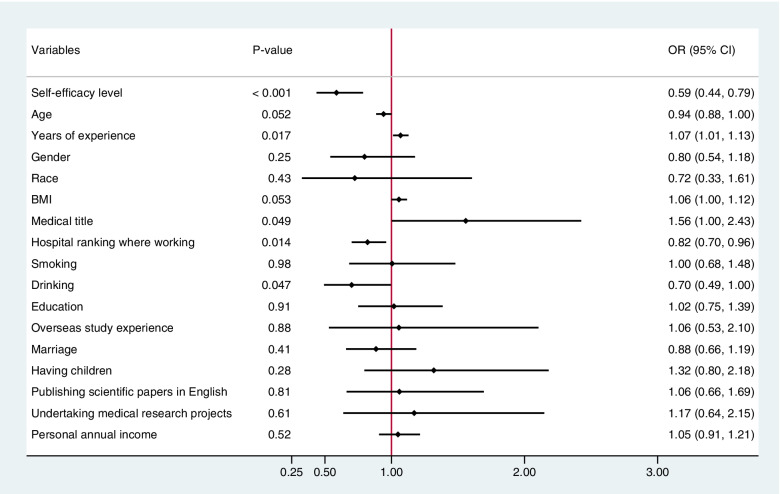
The first logistic regression model of factors correlated with ARAEs. BMI, body mass index; ARAEs, anesthesia-related adverse events; OR, odd ratio; 95% CI, 95% confidence interval. Univariate logistic regression analysis was used to identify the factors correlated with ARAEs

**Fig. 3:**
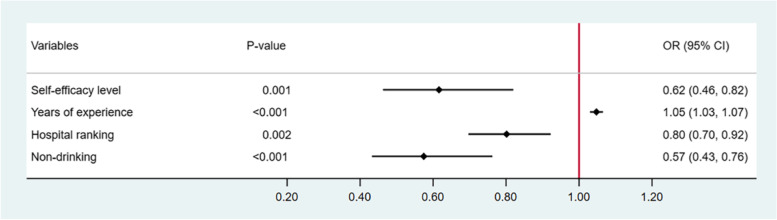
The final logistic regression model of factors correlated with ARAEs. ARAEs, anesthesia-related adverse events; OR, odd ratio; 95% CI, 95% confidence interval

The first logistic regression model was included some variables, as shown in Fig. 2. In the final multivariable logistic regression model, self-efficacy level (OR, 0.62; 95% CI, 0.46–0.82; *P* = 0.001), years of experience (OR, 1.05; 95% CI, 1.03–1.07; *P* < 0.001), hospital ranking (OR, 0.8; 95% CI, 0.70–0.92; *P* = 0.002), and non-alcohol drinker (OR, 0.57; 95% CI, 0.43–0.76; *P* < 0.001) were significantly correlated with ARAEs, as shown in Fig. 3. When controlling other covariates of years of experience, non-alcohol drinker and hospital ranking, the odds ratio of ARAEs was 0.62 in anesthesiologists with high level self-efficacy than in anesthesiologists normal level self-efficacy.

## Discussion

To our knowledge, this observational survey was the first large-scale study to investigate an association between perceived self-efficacy and ARAEs in anesthesia community. The clinically meaningful and statistically significant correlation between the self-efficacy level and ARAEs was found in this work. Logistic regression analysis demonstrated that self-efficacy was also associated with ARAEs. Therefore, lower level self-efficacy was a risk factor for ARAEs during clinical work.

Previous studies observed and described the value and effect of self-efficacy in healthcare professionals [16, 27]. Our findings also provided strong evidence that perceived self-efficacy was apparently associated with ARAEs among anesthesiologists. Anesthesiologists with high level of perceived self-efficacy had fewer accidents during anesthesia practice. In our study, perceived self-efficacy was measured through self-report, reflecting the all-around and comprehensive judgment of anesthesiologists regarding the level of clinical skills acquired and the strength of that belief. Thus, we speculated that a high level of self-efficacy, as a self-affirmation of clinical techniques, may promotes an increased sense of self-identity and self-confidence and positively affects clinical work and practice.

However, perceived self-efficacy means someone feels that he/she has the ability to successfully execute a skill or behavior, even if he/she has never done it before, which slightly differs from self-confidence. Self-confidence, a psychological indicator of evaluating self-competence, grows and alters with previous experiences or work. Elfenbein DM et al. have also described that self-efficacy is the social phenomenon shaped and developed not by the impersonal acquisition of skills and technical expertise but also by the absorption of the views and attitudes of others [16]. Whereas confidence is individually and subjectively understood and interpreted. According to the psychologist Albert Bandura, “Confidence is a nonspecific term that refers to strength of belief but does not necessarily specify what the certainty is about. Confidence is a catchword rather than a construct embedded in a theoretical system.” [28]. Hence, we argued that self-confidence was a general and colloquial term and can be closely described by self-efficacy to some extent.

We found that a high level self-efficacy was associated with a decreased incidence of ARAEs. Previous studies have also addressed that high level self-efficacy reduced socioeconomic inequalities in emotional symptoms [29], correlated with lower odds of pre-frailty/frailty in older adults with chronic disease [30], improved well-being and quality of life in cardiac patients [31]. This evidence stressed benefits from high level self-efficacy in different conditions. Nevertheless, Pena G et al. insist that overinflated self-efficacy may have deleterious consequences to patients. Clinicians with inflated self-efficacy might make mistakes, as they fail to consider options due to overconfidence. Firstly, we affirmed that overconfidence is not equal to inflated self-efficacy, as both are essentially different. Secondly, there is considerable difficulty in defining the concept of inflated self-efficacy, which is short of objective criteria or reverences. At last, inflated or high self-efficacy is harmful for performing a specific behavior, which lacks empirical evidence.

Since the middle of the twentieth century, considerable interest has been attracted to investigate self-efficacy in professional and academic areas. In the literature reviewed, few studies have aimed to examine the predictive effect of self-efficacy on adverse events and accidents produced by clinicians. However, there are still some related investigations from different approaches that address self-efficacy as a feasible predictor of guideline adherence [32], clinical decision-making [33], and communication skills [34]. Furthermore, an interview study enrolled 28 physicians and reported that self-doubt is detrimental to clinicians [35]. In contrast, this paper offers new data and predictive value that self-efficacy has important clinical practical implications.

In addition, years of experience, hospital ranking, and non-alcohol drinker were significantly correlated with ARAEs. The underlying mechanisms remain to be elucidated. Obviously, in lower-grade hospitals, anesthesia-related operations or practices are not complicated as in high-grade hospitals, which possibly decreases the incidence of ARAEs. Regarding years of experience, elderly anesthesiologists have more chance to experience ARAEs under a long time clinical anesthesia work when compared to the young. Interestingly, drinking can cause more ARAEs. Maybe, it can potentially be explained by the fact that drinking might trigger medical, psychiatric, and behavior-related complications and risks [36–38]. Therefore, drinking-related dysfunction in the central nervous system and health impairment may partly disturb emergency response capacity and clinical decisions, contributing to ARAEs. These explanations and reasoning are derived from documented studies and remain to be validated in future work.

This study has some important strengths. First, we addressed the association between perceived self-efficacy and ARAEs. To the best of our knowledge, this is the first large-sample study on this subject to employ ideal scales to assess the association between self-efficacy and ARAEs. Second, we also systematically examined several factors covering demographics, academic performance, medical work information, and self-evaluation, which were potentially correlated with ARAEs. Third, the self-efficacy scale used in the present paper has a high level of internal consistency, reliability, and validity, and captures more accuracy and credibility than other scales [39]. Finally, both propensity score matching and multivariable logistic regression were utilized to examine the composite of ARAE, which improves the efficacy in statistics.

The underlying limitations of this study need to be illustrated. Our survey-based investigation using a cross-sectional study design recorded both self-reported and self-measured data, in part resulting in response bias. This study further controlled the bias by expanding the sample size, using multivariate analysis, and propensity score matching. Furthermore, this is an observational study, which may be subject to unmeasured confounding such as burnout. Burnout was reported to be associated with both physicians’ self-reported errors and perceived self-efficacy, and a high level of self-efficacy can ameliorate self-reported errors and job burnout [40, 41]. The relationship between self-efficacy and burnout might be mutual influence and interaction, which may be involved in causing ARAEs. Our future work will focus on the effect of mutual interaction of both on anesthesiologists’ self-reported errors.

At first, Bandura’s social cognitive theory was much sophisticated, which partly lead to difficulty in fully interpret his grand theory in the present study even with the help of a psychologist. Additionally, we did not evaluate anesthesia-related technical skills (tracheal intubation, regional nerve block, induction of anesthesia, etc.) via this online questionnaire and their correlation with ARAE. We considered that the questionnaire-documented self-reported data might lead to great measurement bias, which is unfit for the assessment of technical skills. It is reasonable that clinical technical assessment be conducted through practical tests rather than self-report questionnaires.

## Conclusions

Our study reports a comprehensive assessment of risk factors for ARAEs. Our results found a clinically meaningful and statistically significant correlation between the self-efficacy level and ARAEs. These findings in part, support that clinical educators and hospital managers should pay more attention to self-efficacy of practitioners.

## Supplementary Information

**Additional file 1: Table-S1** Introduction of anesthesia-related adverse events (ARAEs).

## Data Availability

The datasets used and/or analysed during the current study available from the corresponding author on reasonable request.

## Abbreviations

ARAEs: Anesthesia-related adverse events
SD: Standard deviation
IQR: Interquartile range
BMI: Body mass index
CI: Confidence interval
RR: Relative risk
RD: Risk difference
aRR: Adjusted relative risk
aRD: Adjusted risk difference
OR: Odds ratio
KMO: The KMO measure of sampling adequacy
DW: Durbin-Watson test
VIF: Variance inflation factor
CAA: Chinese Association of Anesthesiologists
