# Supplementary material for: The role of anesthesiologists’ perceived self-efficacy in anesthesia-related adverse events
Source: BMC Anesthesiol. 2022 Jun 20;22:190. doi: 10.1186/s12871-022-01732-3 (PMC9208201; doi:10.1186/s12871-022-01732-3)
Supplement: Supplementary file 1 — Additional file 1: Table-S1 Introduction of anesthesia-related adverse events (ARAEs). [file 12871_2022_1732_MOESM1_ESM.docx]

| Table-S1. Type and number of anesthesia-related adverse events (ARAEs) | | |
| --- | --- | --- |
| No. of ARAEs | | Type of anesthesia-related adverse events |
| Normal level group (178) | High-level group (118) |  |
| 20 | 11 | airway injury |
| 14 | 8 | nervous system injury, other injuries |
| 7 | 7 | airway management difficulties |
| 1 | 0 | intraoperative awareness |
| 9 | 7 | cardiac arrest |
| 18 | 11 | severe hypotension/hypertension |
| 8 | 11 | cardiocerebral events |
| 11 | 7 | severe hypoxia |
| 9 | 11 | life-threatening arrhythmia |
| 7 | 4 | severe allergy |
| 10 | 7 | blood transfusion complications |
| 1 | 0 | malignant hyperthermia |
| 6 | 4 | medication events |
| 0 | 1 | perioperative deaths |
| 14 | 2 | respiratory events |
| 19 | 11 | equipment issues |
| 9 | 7 | wrong sided procedures |
| 1 | 1 | severe complications caused anesthesia procedures (wrong medication) |
| 14 | 8 | other anesthesia-related accidents inducing damage to patients |
